# A highly transparent and ultra-stretchable conductor with stable conductivity during large deformation

**DOI:** 10.1038/s41467-019-11364-w

**Published:** 2019-07-31

**Authors:** Zhouyue Lei, Peiyi Wu

**Affiliations:** 1 State Key Laboratory for Modification of Chemical Fibers and Polymer Materials, College of Chemistry, Chemical Engineering and Biotechnology, Center for Advanced Low-Dimension Materials, Donghua University, Shanghai, 201620 China; 2 State Key Laboratory of Molecular Engineering of Polymers, Department of Macromolecular Science and Laboratory for Advanced Materials, Fudan University, Shanghai, 200433 China

**Keywords:** Polymers, Sensors and biosensors

## Abstract

Intrinsically stretchable conductors have undergone rapid development in the past few years and a variety of strategies have been established to improve their electro-mechanical properties. However, ranging from electronically to ionically conductive materials, they are usually vulnerable either to large deformation or at high/low temperatures, mainly due to the fact that conductive domains are generally incompatible with neighboring elastic networks. This is a problem that is usually overlooked and remains challenging to address. Here, we introduce synergistic effect between conductive zwitterionic nanochannels and dynamic hydrogen-bonding networks to break the limitations. The conductor is highly transparent (>90% transmittance), ultra-stretchable (>10,000% strain), high-modulus (>2 MPa Young’s modulus), self-healing, and capable of maintaining stable conductivity during large deformation and at different temperatures. Transparent integrated systems are further demonstrated via 3D printing of its precursor and could achieve diverse sensory capabilities towards strain, temperature, humidity, etc., and even recognition of different liquids.

## Abstract (web summary)

To realize the potential of stretchable conductors for next-generation applications, the compatibility between the conductive and elastic material phases must be improved. Here, the authors report a stretchable conductor with molecular synergy between its conductive and stretchable components.

## Introduction

Soft conductive materials help to bridge the gap between human and machine^1–3^, and bring revolution for artificial intelligence and biological systems^4,5^. Traditionally, there have been two major strategies to render materials not only conductive but also soft and stretchable. One is to introduce soft regions to modify the morphology of conjugated conductive polymers^6,7^, by adding plasticizers^8^ or flexible segments^9^. The other method is to blend conductive components, such as electronically conductive fillers^10^ or ionic electrolytes^11,12^, with insulating stretchable networks. The essential issue is how to manage the conductive paths and the insulating networks.

However, for the majority of current conductors, the compatibility between conductive domains and neighboring elastic networks remains a problem that is usually overlooked, challenging to address, and always leads to poor electro-mechanical performance. For example, physically and/or chemically modified conductive polymers show limited stretchability as a result of the intrinsic incompatibility of their rigid conjugated structures and flexible domains. As for insulating stretchable polymers blended with electronically conductive fillers, such as inorganic particles^10,13^, rigid conjugated polymers^14^, or liquid metals^15^, these immiscible fillers tend to aggregate and result in percolating conductive paths of hundreds of nanometers or even larger sizes, severely impede adaptive movement of the conductive networks during deformation, and often cause irreversible damage of the electronic properties after stretching. In recent years, ionic electrolytes with better affinity to polymers have also been used as conductive components, including aqueous solutions of inorganic salts^11,16–19^, ionic liquids (ILs)^12,20,21^, and electrolyte salts^22^. Different from electronically conductive composites, the ionic electrolytes in these ionic conductors are dominant as the solvents, while the single-network or double-network polymers are embedded in them. This feature guarantees the continuity of the conductive phase during deformation, but on the other hand, too much solvated ionic electrolytes fails to be effectively bonded by polymer networks and thus give rise to the drawback of environmental instability, i.e., potential leakage during deformation, evaporation at high temperatures, and freezing at low temperatures^5,23^.

Inspired by both advantages and drawbacks of current strategies, we believe it is important to fabricate soft conductive nanochannels and couple them with dynamically crosslinked networks via molecular synergy. Herein, we introduce a type of intrinsically stretchable conductors. Small-molecular liquid-like electrolytes, such as ILs, provide charge carriers; polymers with similar ionic structures, e.g., polyzwitterions, realize ionic synergy with the liquid electrolytes and thus assemble conductive nanochannels to avoid aggregates or leaking risk of the electrolytes; dynamic networks are constructed by polymers that also have molecular synergy with the conductive nanochannels, to guarantee the structural integrity, deformation adaptability and environmental stability.

## Results

### Molecular synergistic design

**Fig. 1:**
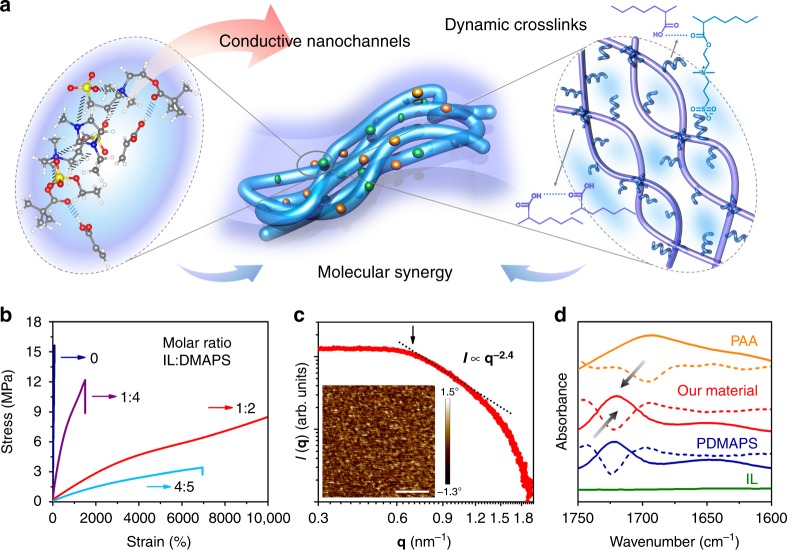
Molecular synergistic design. **a** Schematic illustration of the molecular synergistic design, including the optimized ion-rich structure predicted by DFT and the dynamic hydrogen-bond networks. **b** The true tensile stress–strain curves of the conductors with different contents of the IL. The strain rate is 0.17 s^−1^. **c** The SAXS profile of the intrinsically stretchable conductor. The inset picture is the AFM phase image of the conductor. (scale bar: 100 nm) **d** IR spectra and corresponding second derivative curves of the intrinsically stretchable conductor, PAA, PDMAPS, and IL in the region of 1750–1600 cm^−1^

The schematic illustration of the material design is shown in Fig. 1a. We use a well-known polyzwitterion^24^, i.e., poly(3-dimethyl(methacryloyloxyethyl) ammonium propane sulfonate) (PDMAPS), to assemble ion-rich nanochannels with an IL (1-ethyl-3-methylimidazolium ethyl sulfate) and meanwhile couple them with hydrogen-bonding networks. Since the polyzwitterion is a potential hydrogen-bond acceptor (with ester carbonyl groups), thus a hydrogen-bond donor, e.g., poly(acrylic acid) (PAA) (with carboxylic acid groups), is chosen for fabricating the dynamic hydrogen-bonding networks. Compared with other sorts of polyzwitterions and ILs, such as polycarboxybetaines, polyphosphobetaines, ammonium‐type, chloride-based ILs, etc., the zwitterionic motifs of PDMAPS with sulfonate show closer affinity to the ILs with sulfate. Therefore, we choose this ternary system as a proof-of-concept demonstration of our molecular synergistic design. Nevertheless, we believe the design principles shed light on the preparation of a variety of intrinsically stretchable conductors.

The ionic synergy in the conductive regions are predicted by density functional theory (DFT) calculations at the ωB97X-D level using the 6–31 g(d) basis set (Fig. 1a, Supplementary Fig. 1, and Supplementary Note 1). The attractive binding energies resulting from Coulomb potential inside the polyzwitterion and the IL are 28.6 and 83.1 kcal mol^−1^, respectively. When these four ionic moieties are mixed together, the hybrid system further introduces Coulomb potential between the polyzwitterion and the IL. The attractive binding energy for the ion-rich domain is reduced to 9.6 kcal mol^−1^, which not only favors ionic synergy through ion-dipole interactions between the polyzwitterion and the IL, but also enhances segmental motions of the polyzwitterion. With the incorporation of PAA, the overall attractive binding energy is 14.4 kcal mol^−1^. This material takes an optimized structure, in which one unit of the IL interacts with two monomer units and hydrogen bonds form between PAA and PDMAPS.

The optimized ion-rich structure shows very impressive mechanical properties. In Fig. 1b, when the molar ratio of the IL and DMAPS is varied from 0, 1:4, 1:2 to 4:5, the stretchability and modulus of these conductors significantly change in a wide range. Without the IL, strong internal dipole–dipole interactions from ion-rich domains of polyzwitterion lead to high modulus and low stretchability. Upon the addition of the IL, the modulus decreases accompanied with the increase of the stretchability, probably due to the fact that Coulomb potential inside the polyzwitterion decreases and the formation of soft ion domains with decreased energy barriers. However, with the excess IL, (e.g., when the molar ratio reaches 4:5), the polyzwitterion fails to effectively bind the IL, similar to the previously reported ionogels or hydrogels, leading to low modulus, low stretchability and potential leakage of the ionic electrolyte. Obviously, the optimal structure is shown by the sample with the molar ratio of 1:2, which is in good agreement with the DFT prediction. Therefore, we choose this sample for further characterizations.

The nanostructure of this intrinsically stretchable conductor is further revealed by the small angle X-ray scattering (SAXS) profile and the atomic force microscope (AFM) phase image in Fig. 1c. In the Porod regime, *I*(***q***) is given by the power-law equation, $I\left( {\mathbf{q}} \right) \propto {\mathbf{q}}^{ - z}$, where the exponent *z* is determined as 2.4. It indicates there is mass fractal and the ion-rich domains are dispersed as interconnected (branched) networks, confirming the formation of ion channels in our material^25^. Besides, the scattering shoulder at low scattering vectors, as indicated by an arrow, suggests long-range order of the ion channels^26^. The mesh size of these channels is estimated as $\frac{{2{\mathrm{\pi }}}}{{\mathbf{q}}}$, i.e., about 10-nm diameter. The SAXS result is verified by the AFM phase image, which clearly shows the soft ion-rich domains (dark regions) not only are well confined in the dynamic networks (bright regions) but also form the interconnected conductive networks. This phase separation nanostructure also looks similar to the nanofibril structure reported in a stretchable and highly conductive PEDOT:PSS hydrogel^27^. Both of them form interconnected conductive channels. The PEDOT:PSSS hydrogel shows much higher conductivity owing to the ordered assembly of PEDOT-rich crystallines, while our material exhibits higher stretchability because the dynamic networks are composed of amorphous flexible polymers. However, as a contrast in Supplementary Fig. 2, without the IL, there is no significant phase separation observed either in the AFM image or the SAXS plot, which indicates a very uniform structure and the mesh size is very tiny. It supports the DFT prediction and also explains the reason for the high modulus and low stretchability. With a small amount of IL (the molar ratio of IL and DMAPS is 1:4), slight phase separation is observed in the AFM image. This is in line with its SAXS profile. In the Porod regime, the exponent *z* is determined as 2.0, indicating the preliminary formation of mass fractal structures. The scattering shoulder, as indicated by an arrow, suggests the phase separation on the order of 7 nm. However, with much more IL (the molar ratio of IL and DMAPS is 4:5), the IL acts like the solvents and the polymers are uniformly dispersed in them. We could not find either phase separation nanostructure in the AFM phase image or scattering shoulder on the order of nanometers in the SAXS plot. This nanostructure also supports the above discussion that too much IL results in gel-like low modulus.

The hydrogen-bonding synergy between the ion nanochannels (i.e., conductive paths) and dynamic networks is confirmed by FTIR results (Fig. 1d and Supplementary Fig. 3). The strong hydrogen bonds, e.g., cyclic hydrogen bonds from carboxylic acid groups of pure PAA with dimeric form, are located at very low frequency, i.e., at 1695 cm^−1,^^28^, while the free ester carbonyl groups in PDMAPS are located at 1724 cm^−1^. In our material, the stretching band of carbonyl groups shifts to 1721 cm^−1^, which indicates the formation of weak hydrogen bonds between the hydrogen-bond acceptors and hydrogen-bond donors.

### Mechanical properties

**Fig. 2:**
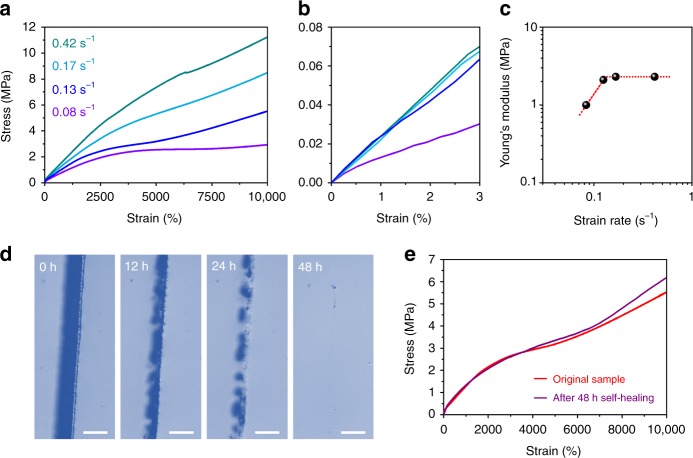
Mechanical properties. **a** True tensile curves of the intrinsically stretchable conductor at different strain rates. **b** True tensile curves of the conductor at small strain. **c** Young’s modulus of the intrinsically stretchable conductor at different strain rates. **d** Optical micrographs record the autonomously self-healing process of the conductor (scale bar: 100 μm). **e** True tensile stress–strain curves of the original and self-healing conductor

As a result of the rational design of molecular synergy, the conductor exhibits impressive mechanical properties. As shown in Fig. 2a, it could achieve an ultra-high elongation-at-break beyond 10,000% strain in a wide range of deformation rates from 0.08 to 0.42 s^−1^. The Young’s modulus increases from 1 MPa at small strain rates <0.14 s^−1^, and saturates at 2.3 MPa at high strain rates (Fig. 2b, c). Such impressive mechanical performance has been rarely reported in traditional electronically or ionically conductive materials, and is ascribed to the molecular synergy between soft conductive nanochannels and dynamic crosslinks based on hydrogen bonds. We could roughly estimate the lifetime of the hydrogen bonds according to the critical strain rate 0.14 s^−1^ shown in Fig. 2c^29–31^. It is larger than the reciprocal of the average lifetime of stronger amide–amide hydrogen bonds, thus indicating lower bond strength^32,33^. Therefore, without permanently crosslinked networks, the weak hydrogen bonds not only contribute to relatively high modulus during deformation, but also allow relaxation of the dynamic networks in a relatively long time scale, which is beneficial to ultra-high stretchability, autonomous self-healing, and synergistic movement of the conductive paths during large deformation. The self-healing process is recorded by time-dependent optical micrographs (Fig. 2d). Within 48 h, a crack between two fractured materials disappears and the autonomously self-healing sample shows similar ultra-high stretchability beyond 10,000% strain (Fig. 2e).

Due to the existence of dynamic networks, the conductor is also elastic and recoverable, as shown in a video of a manual stretch-release process (Supplementary Movie 1 and Supplementary Fig. 4). It could recover to the original shape upon unloading the stress within 70 s. The cyclic stretching measurements further quantitatively assess the recoverability of the polymer networks based on the area ratio of the stress–strain curves. They demonstrate effective energy dissipation with pronounced hysteresis, and the conductor achieves a recovery ratio of 63% after 10 cycles of the continuous stretching to 500% strain and a resting period of 1 h (Supplementary Fig. 5). As the stretching strain increases, the polymer networks are more prone to fatigue, which is also widely observed in chemically crosslinked materials, e.g., Ca-alginate/polyacrylamide hydrogels^34–36^. The macroscopic relaxation time of the conductor is analyzed by the stress-relaxation curves (Supplementary Fig. 6). The relaxation time ($\tau$*) is defined as the time required for *T*/*T*_0_ = 1/e (*T* is the tensile stress). It is 148 s when the conductor is stretched to 500% strain and increases to 188 s at the 1000% strain, comparable to tough elastomers crosslinked by both metal–ligand coordination bonds and hydrogen interactions^37^.

**Fig. 3:**
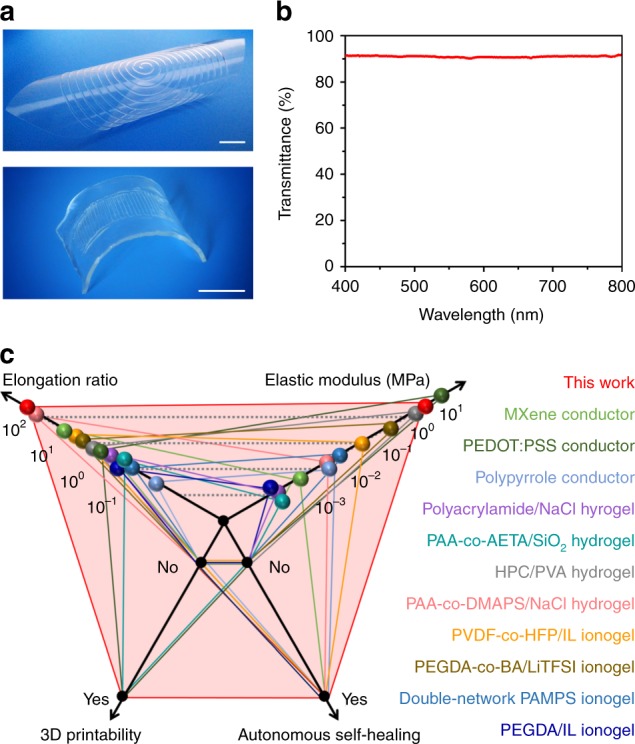
3D printability, transparence, and comparisons. **a** Photographs showing 3D-printing conductors on a polyethylene film (the above) and inside of two VHB elastomers (the bottom) (scale bar: 2 cm). **b** The transmittance of the intrinsically stretchable conductor with a thickness of 0.1 mm in the visible wavelength range of 400–800 nm. **c** A comparison between this work and previously-reported intrinsically stretchable conductors in terms of stretchability, modulus, self-healability and 3D printability^8,11,12,18,20–22,40–42,44^

Another advantage of our material is the 3D printability (direct ink writing) of its precursor. As shown in Supplementary Fig. 7, its precursor aqueous solution is a type of shear-thinning power-law liquid (Supplementary Note 2)^38,39^. Therefore, the precursor can be facilely processed and directly printed on different substrates to construct multi-layer structures (Fig. 3a). Besides, it is also highly transparent. For a sample with a thickness of 0.1 mm, the transmittance achieves up to 90.7% at 550 nm and it is fully transparent in visible light region (Fig. 3b). The high-transparence results from the homogeneous microstructures and the molecular synergy in and along with the conductive nanochannels. This is distinct from traditional electronically conductive materials that are composed of large-size aggregates beyond hundreds of nanometers and thus show decreasing transparency^10,13^.

Compared with current intrinsically stretchable conductors, this material shows extraordinary mechanical versatility in terms of ultra-stretchability, high modulus, autonomous self-healing, and 3D printability. It is well-known that, with the improvement of self-healing efficiency and elongation ratio, materials’ elastic modulus usually decreases. Besides, it is difficult for electronically conductive materials with conjugated structures or nanofiller aggregates to achieve the stretchability beyond 1000% strain^8,40,41^. To the best of our knowledge, even for ionically conductive materials based on hydrogels or ionogels, they have been rarely reported for an elongation-at-break beyond 10,000% strain while maintaining a modulus of >2 MPa as well as autonomous self-healing^11,12,18,20–22,42–44^. Therefore, this material with the combination of ion nanochannels and dynamically crosslinked networks, takes a step forward in the optimization of the mechanical properties of intrinsically stretchable conductors. A detailed comparison is shown in Fig. 3c.

### Electrical properties

**Fig. 4:**
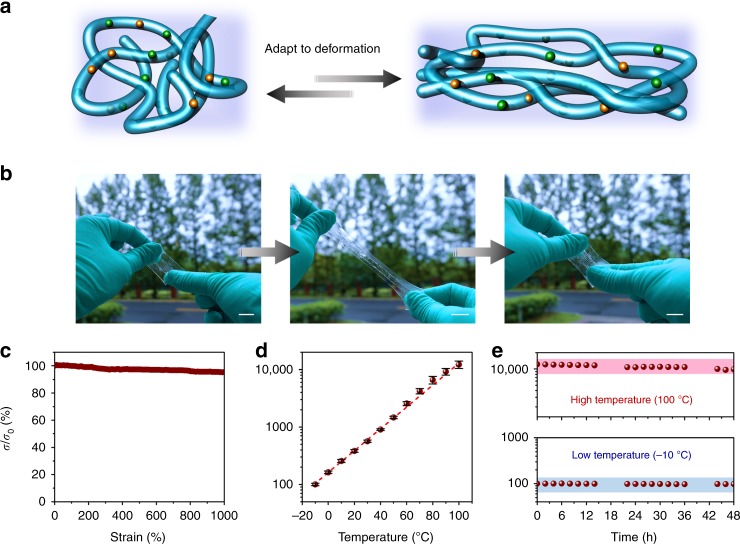
Electrical properties in dynamic environments. **a** Schematic illustration of the conductive paths in this material adapting to deformation. Blue lines represent ion nanochannels and the purple parts represent the dynamic networks. **b** Photographs of the 3D-printing conductor during a stretch-release cycle (scale bar: 2 cm). **c** The stability of the ionic conductivity during the deformation process. **d** The relative changes in ionic conductivity in the temperature range of −10 to 100 °C (error bars: standard deviations). **e** The stability of the ionic conductivity at extreme low or high temperatures for a long period. These measurements are humidity control (60 RH%)

The conductivity of this material is about 1 × 10^−2^ S m^−1^ at ambient condition (25 °C, 60% RH), verified by different measuring methods (Supplementary Note 3 and Supplementary Fig. 8). As illustrated in Fig. 4a, this conductor can store, generate, and transmit electrical signals via the spatial flow and temporal distribution of ions and ion nanochannels. The conductive paths could dynamically adapt to deformation. For example, when the material is printed on a high-adhesion dielectric elastomer (VHB 4905, 3M), it is not only compliant with the substrate during the dynamic deformation (Fig. 4b), but also shows highly stable conductivity with large strains (Fig. 4c). This is different from the conductors based on conductive percolating paths with large-size aggregates, which usually fall apart at large deformation (Supplementary Note 4). As compared in Supplementary Fig. 9, an electronic conductor, whose conductive networks are fabricated by graphene fillers with 1 μm diameter, quickly breaks at 71% strain.

This material is also quite stable in ambient condition with only about 3% change of its conductivity in 24 h (Supplementary Fig. 10a). In contrast, an ionogel which is composed of only PAA and the same IL shows a decrease of 33.7% of the conductivity after 24 h, probably owing to the weak bonding between the IL and the polymer networks. (Supplementary Fig. 10b). As for hydrogels, whether chemically crosslinked hydrogels or physically crosslinked hydrogels, their conductivity reduces by more than 99% in ambient condition because of the rapid loss of the free water (Supplementary Fig. 10c, d). Squeezing tests further suggest the instability of the solvated conductive electrolytes in the traditional ionic conductors but the effective confinement of the IL in our material. When we squeeze the PAA/IL ionogel, the IL leaks on a plain paper (Supplementary Fig. 11 and Supplementary Movie 2). The similar phenomenon is also observed in a polyacrylamide/NaCl hydrogel with the leakage of free water (Supplementary Fig. 12 and Supplementary Movie 3). Fortunately, when we squeeze the conductor reported in this work, there is no leaking liquid on the plain paper (Supplementary Fig. 13 and Supplementary Movie 4).

Under extremely high or low-temperature environments, there is no melting point or glass transition observed in this conductor from its differential scanning calorimetry (DSC) curves. The polymer networks will neither be completely frozen at sub-zero temperatures nor be destroyed at 100 °C, as further confirmed by the dynamic mechanical analysis (DMA) and rheological behaviors (Supplementary Note 5 and Supplementary Figs. 14 and 15)^45,46^. When temperature increases, the ionic conductivity of the intrinsically stretchable conductor is improved, and vice versa. This is due to the fact that different temperatures contribute to different kinetic energies of charge carriers, which is also a phenomenon widely observed in electronic conductors^47,48^ but distinct from the breakdown of the conductive networks owing to the loss of ionic electrolytes^18^. In a logarithmic coordinate system, there is an almost linear relationship between the relative conductivity (*σ*/*σ*_0_) and temperature (Fig. 4d). The material’s conductivity adapts to the temperature changes at first and then maintains a good stability for a long period (Fig. 4e). Overall, because the ion nanochannels are adaptable to deformation and confined in the dynamic networks, this conductor can not only avoid the disconnection of conductive paths and the leakage of electrolytes during deformation, but also show anti-evaporation at high temperatures and anti-freezing at low temperatures.

### Transparent integrated deformable sensory systems

**Fig. 5:**
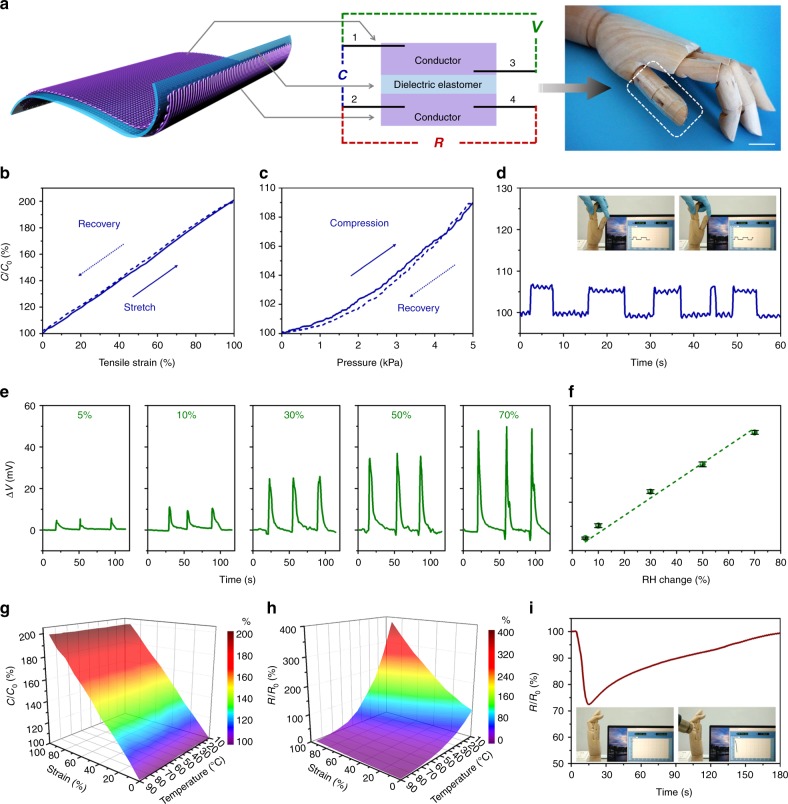
An integrated sensory system. **a** The 3D-pringting architecture, circuit schematic illustration and a photograph of an integrated transparent sensory system based on the intrinsically stretchable conductor (scale bar: 2 cm). **b** Capacitance-strain cycling curves of the sensory system. **c** Capacitance-pressure cycling curves of the sensory system. **d** The sensory system tracks a robotic hand’s movements. Inset photographs are from Supplementary Movie 5. **e** Voltages generated by environmental humidity changes (relative humidity, RH). **f** The sensory system can perceive environmental RH changes based on voltage changes (error bars: standard deviations). **g** The capacitive response of the sensory system upon mutual effect of temperature and strain. **h** The resistive response of the sensory system upon mutual effect of temperature and strain. **i** The sensory system detects environmental temperature changes caused by a thermal lance *via* resistance signals. Inset photographs are from Supplementary Movie 6

With a multi-layer circuit design, this intrinsically stretchable conductor can be integrated in deformable sensory systems for future soft robotic applications. As shown in Fig. 5a, the intrinsically stretchable conductor is directly printed on a dielectric elastomer (VHB) with a triple layer structure. After being dried for at least a week, the uppermost and lowermost conductors are connected to four metal electrodes, and the intermediate dielectric elastomer allows charge carriers to be stored in the conductive layers. Parallel-plate capacitance of the whole sensory system can be read from the electrodes 1 and 2 which indicates mechanical stimuli^16^. The uppermost layer senses humidity changes from moisture-driven ion flows between electrodes 1 and 3^49^. The intrinsically stretchable conductor at the lowermost layer is applied to monitor temperature changes based on a temperature-resistance relationship recorded by electrodes 2 and 4. Therefore, this intrinsically stretchable conductor can fabricate a transparent integrated system to simultaneously mimic mechano-receptor, humidity-receptor and thermo-receptor of natural skins, and may provide a wide range of sensory capabilities for soft robots.

The transparent sensory system could report mechanical stimuli including stretching, compressing and bending by using capacitance signals, as shown in Fig. 5b–d. It shows reversibly linear relationship between strain and capacitance with a stable sensitivity of about 100% (Fig. 5b), in accordance with a theoretical prediction of *C* = *C*_0_*λ* (*λ* is the strain factor and *C*_0_ is the initial capacitance without deformation, and more details are available in Supplementary Note 6)^16^. Upon compression, this system shows reversible capacitance changes which are supposed to be related with the modulus of the dielectric layer in theory (Fig. 5c, and more details are available in Supplementary Note 7). When this transparent sensory system attaches to a robotic hand, it is mechanically adaptable to the hand’s movements and can track the movements with real-time capacitance signals (Fig. 5d, Supplementary Movie 5). Besides functioning as artificial mechano-receptor, it can sense humidity changes through self-powered voltages at the uppermost layer. Since the polyzwitterion is hydrophilic, more ions will be released upon exposing to moisture. When environmental humidity changes, it leads to humidity difference between the electrodes 1 and 3 and initiates an ion concentration gradient. The ion concentration gradient results in a self-powered voltage as shown in Fig. 5e (more details are available in Supplementary Note 8). The system shows a baseline at 30% relative humidity (RH) and outputs different voltages upon exposing to different humidity. A linear relationship between the humidity changes and the output voltages with a high sensitivity up to 0.72 is observed, making it convenient for humidity sensing (Fig. 5f). Meanwhile, the conductor at the lowermost layer is applied to monitor temperature changes. Its ionic conductivity is independent of environmental humidity changes since it is isolated from air. In addition, the “capacitance vs strain vs temperature” space further isolates the strain-induced resistance changes (Fig. 5g, Supplementary Fig. 16) and makes it possible to derive the temperature changes from the “resistance vs strain vs temperature” space. Here we show the robotic hand attached with the transparent sensory system can detect environmental temperature changes induced by a thermal lance. The resistance quickly decreases when the robotic hand is heated and recovers to its initial state upon removing the heating source (Fig. 5i and Supplementary Movie 6).

**Fig. 6:**
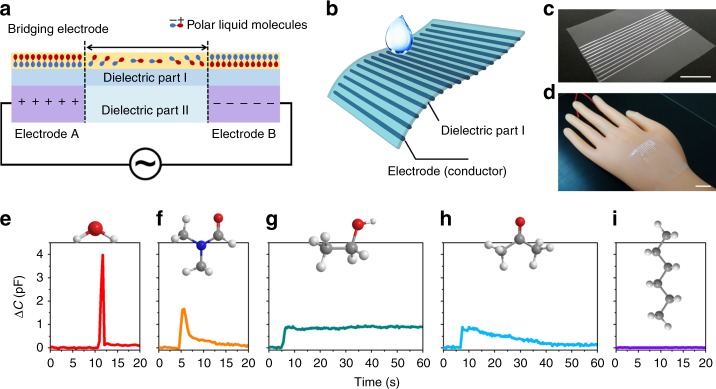
Recognition of different liquid molecules. **a** The circuit schematic illustration of a sensory system to identify different liquid molecules. **b** The 3D-pringting architecture of the sensory system. **c** A photograph of the 3D-pringting sensory system (scale bar: 2 cm). **d** A photograph of the 3D-pringting sensory system attached to a robotic hand (scale bar: 2 cm). **e**–**i** Different capacitive responses when different liquids, i.e., **e** water, **f** DMF, **g** ethanol, **h** acetone, **i** n-hexane, drip on the robotic hand covered with the sensory system

In addition to the common sensory capabilities found in natural skins such as strain, stress, humidity, and temperature, the intrinsically stretchable conductor can also fabricate sensory systems with the functions beyond natural skins. Here a 3D-printing electrode array of the intrinsically stretchable conductor is demonstrated with the ability to identify different liquid molecules according to their different polarity and surface tension. When any two of the 3D-printing parallel electrodes are connected to an alternating current (AC) power source, this circuit shows negligible capacitance in the resting state, as shown in Fig. 6a, b. When a polar liquid falls on the upper dielectric layer, it forms a bridging electrode owing to the polarization of the liquid in AC electric field, which thus leads to increasing capacitance signals between the electrodes A, B (the conductor) and the bridging electrode (the polar liquid)^50^. On the basis of this mechanism, we print the intrinsically stretchable conductor on a dielectric polyethylene layer and attach it to a robotic hand (Fig. 6c, d). This sensory system allows the robotic hand to recognize different types of liquid molecules owing to their different polarity, volatility, as well as wettability to the dielectric polyethylene layer. The polarity of the liquid molecules determines the intensity of the increasing capacitance, while the volatility and wettability are related with duration time of the increasing capacitance. For example, when a polar liquid, e.g., deionized water, with a high dielectric constant (*K*) but low wettability to the non-polar polyethylene layer, drips on the surface, it is easily polarized but quickly slides down, and thus results in a large but short-time increase of the capacitance signals (Fig. 6e). N,N-Dimethyl formamide (DMF), ethanol and acetone with smaller dielectric constants (more details are available in Supplementary Table 1) lead to smaller capacitance increments when they fall on this sensory system, while a non-polar liquid, e.g., n-hexane, has no contribution to the capacitance (Fig. 6f–i, Supplementary Table 1). Moreover, ethanol and acetone have better wettability to the dielectric layer and therefore show relatively longer duration time of the increasing capacitances. Compared to ethanol, acetone exhibits higher volatility in air, so its capacitance increment fades away more quickly. Therefore, it can help a robotic hand to identify at least five types of liquid molecules. Two example movies are also shown in the Supplementary Movies 7 and 8. This sensory system allows soft robotics to make rough predictions about the physical properties of an unknown liquid by comparing real-time electrical signals. Furthermore, it may also enable time-space-resolved recognition towards complex physical and chemical stimuli with an advanced origami hierarchical array^51^. Overall, this intrinsically stretchable conductor will certainly play a vital role in future soft robotics, with the advantages of good transparence, ultra-stretchability, high modulus, and possibilities to replicate and even transcend sensory capabilities of natural creatures.

## Discussion

This work reports an intrinsically stretchable conductor with molecular synergistic design on its conductive paths and dynamic networks. The nanoscale conductive paths could adapt to large deformation, and avoid directly exposing to external environments. Benefiting from the rational designs, this material not only breaks through the limits encountered in current electronic and ionic conductors, with good transparence, ultra-stretchability, high modulus, reconfiguration of the elastic networks, etc., but also greatly improve the stability in dynamic environments. Transparent integrated systems based on this material are further demonstrated via customized additive manufacturing which could achieve a variety of sensory capabilities towards strain, temperature, humidity, etc., and even recognition of different liquid molecules. In particularly, as far as we know, the ability to recognize different liquids has been rarely reported, and it may contribute to higher intelligence of artificial sensory systems. We believe that current work addresses a long-lasting challenge in the field of intrinsically stretchable conductors, provides general inspiration for the nanostructural designs and device fabrications, and could also promote the development of intelligent robots with the requirements for entire softness, high transparence, multiple sensory capabilities, and environmental stability.

## Methods

### Materials

PAA (average *M*_w_ = 250,000), zwitterionic monomer 3-dimethyl(methacryloyloxyethyl) ammonium propane sulfonate (DMAPS), poly(ethylene glycol) diacrylate (*M*_w_ = 575), acrylic acid, and ammonium persulfate (APS) were purchased from Sigma-Aldrich Co. The IL (1-ethyl-3-methylimidazolium ethyl sulfate) was obtained from TCI Co. (Shanghai). Alginate, CaCl_2_, DMF, ethanol, and n-hexane were purchased from Aladdin Chemical Co. Acetone was purchased from Sinopharm Chemical Reagent Co. The high-adhesion dielectric tape VHB (4905) is purchased from Minnesota Mining and Manufacturing Co., whose full name is “Very High Bond”.

### Preparation of precursors of the conductors

Zwitterionic monomer was firstly mixed with IL and then initiated by APS (0.2 mol%) in PAA aqueous solution. The molar ratio of monomer units of PAA and PDMAPS was fixed at 1:1, and the monomer concentration during polymerization is 30 wt%. The polymerization was proceeded at 70 °C for 6 h. In the final samples after being dried, the molar ratio of PDMAPS, PAA and IL is 1:1:0, 4:4:1, 2:2:1, and 5:5:4. For FTIR analysis, pure PDMAPS was synthesized according to the same method without IL and PAA.

### 3D printing of the intrinsically stretchable conductor

The precursors of the intrinsically stretchable conductor could be used for 3D printing on a 3D Bio-Architect work station (Regenovo) or poured into molds to prepare films with any desired shapes. During 3D printing, the precursor was extruded through a tip needle (0.26 mm diameter) with a speed of 1 mm s^−1^ at 25 °C. After being dried for at least a week, the materials are ready for tests.

### Characterizations

The AFM phase images were obtained on Multimode 8 (Bruker) with the taping mode. SAXS experiment was performed on a France/Xenocs XeUSS2.0 with an X‐ray energy of 70 kV and a wavelength of 1.34 Å at room temperature. IR spectra were recorded by using attenuated total reflectance (ATR) method (a diamond crystal as the window material, Nicolet 6700 spectrometer). The transmittance of the intrinsically stretchable conductor was recorded on a Perkin-Elmer UV–Vis spectrophotometer (Lambda 750). Viscometry was performed on a HAAKE MARS modular advanced rheometer with a 25 mm parallel plate at shear rates ranging from 0.1 to 1000 s^−1^ at 25 °C. Tensile curves were recorded on a universal mechanical test machine (Instron 5966) at ambient condition (25 °C, 60% RH). Since the samples had very large deformation, unless otherwise indicated, here the true stress instead of the nominal stress was used, which was calculated from the product of the nominal stress and the stretch factor based on the assumption that the conductors were incompressible. Cyclic stress–strain curves were recorded at a strain speed of 0.17 s^−1^. Stress-relaxation analysis was performed on the universal mechanical test machine. The stretching rate is 0.17 s^−1^ during the stretching process. The optical micrographs of the self-healing process were recorded on a polarized optical microscope (Leica DM2500P). DSC measurement was performed on a Mettler-Toledo thermal analyzer at a scanning rate of 5 °C min^−1^. DMA was performed on a Mettler-Toledo dynamic thermomechanical analyzer (SDTA861e) using the stretching mode with a frequency of 1 Hz and a heating rate of 3 °C min^−1^.

Capacitance and resistance signals were measured on an LCR meter (TH2830) controlled by a customized LabView program at an AC voltage of 1 V and a sweeping frequency of 1 kHz. Voltage signals were recorded on an electrochemical station (CHI 660E). Temperature and humidity were controlled in a temperature and humidity test chamber (Shanghai Bluepard Instruments, BPHJ-120AF). Unless otherwise indicated, sensing performances were recorded in ambient condition.

## Supplementary information

Supplementary Information

Description of Additional Supplementary Files

Supplementary Movie 1

Supplementary Movie 2

Supplementary Movie 3

Supplementary Movie 4

Supplementary Movie 5

Supplementary Movie 6

Supplementary Movie 7

Supplementary Movie 8

## Data Availability

The data that support the findings of this study are available from the corresponding author upon reasonable request.
