# Supplementary material for: A highly transparent and ultra-stretchable conductor with stable conductivity during large deformation
Source: Nat Commun. 2019 Jul 31;10:3429. doi: 10.1038/s41467-019-11364-w (PMC6668389; doi:10.1038/s41467-019-11364-w)
Supplement: Supplementary file 1 — Supplementary Information [file 41467_2019_11364_MOESM1_ESM.pdf]

A highly transparent and ultra-stretchable conductor with stable conductivity during large deformation

Zhouyue Lei[†‡], and Peiyi Wu[†‡*]

[†]State Key Laboratory for Modification of Chemical Fibers and Polymer Materials, College of Chemistry, Chemical Engineering and Biotechnology, Center for Advanced Low-Dimension Materials, Donghua University, Shanghai 201620, China
[‡]State Key Laboratory of Molecular Engineering of Polymers, Department of Macromolecular Science and Laboratory for Advanced Materials, Fudan University, Shanghai 200433, China
E-mail: wupeiyi@dhu.edu.cn

Supplementary Figures

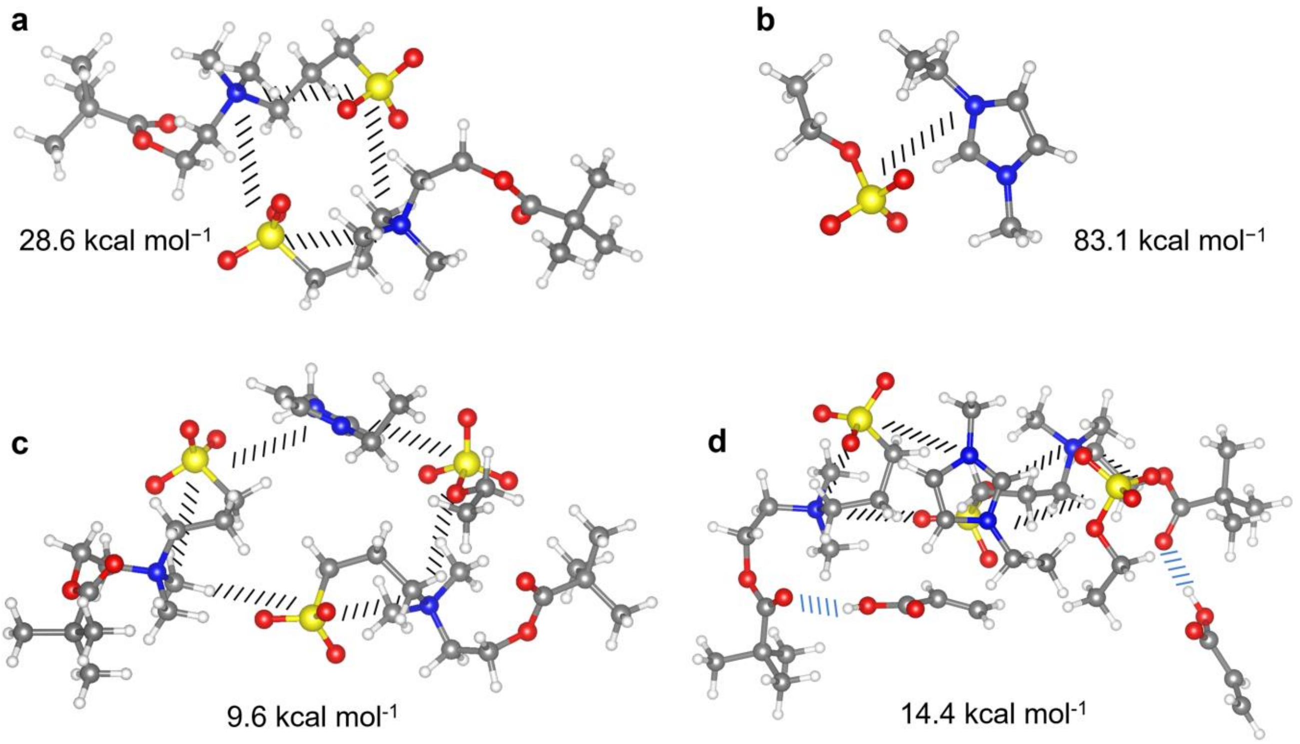

Supplementary Figure 1. DFT calculations of molecule structures and binding energies. (a) Two monomers of PDMAPS interacting *via* dipole-dipole forces with the calculated attractive binding energy of 28.6 kcal mol^{-1}. (b) Intramolecular interaction of the IL with the calculated attractive binding energy of 83.1 kcal mol^{-1}. (c) Two monomers of PDMAPS interact with one unit of the IL, and the attractive binding energy is calculated as 9.6 kcal mol^{-1}. (d) Two monomers of PDMAPS and two monomers of PAA interact with one unit of the IL, and the attractive binding energy is calculated to be 14.4 kcal mol^{-1}.

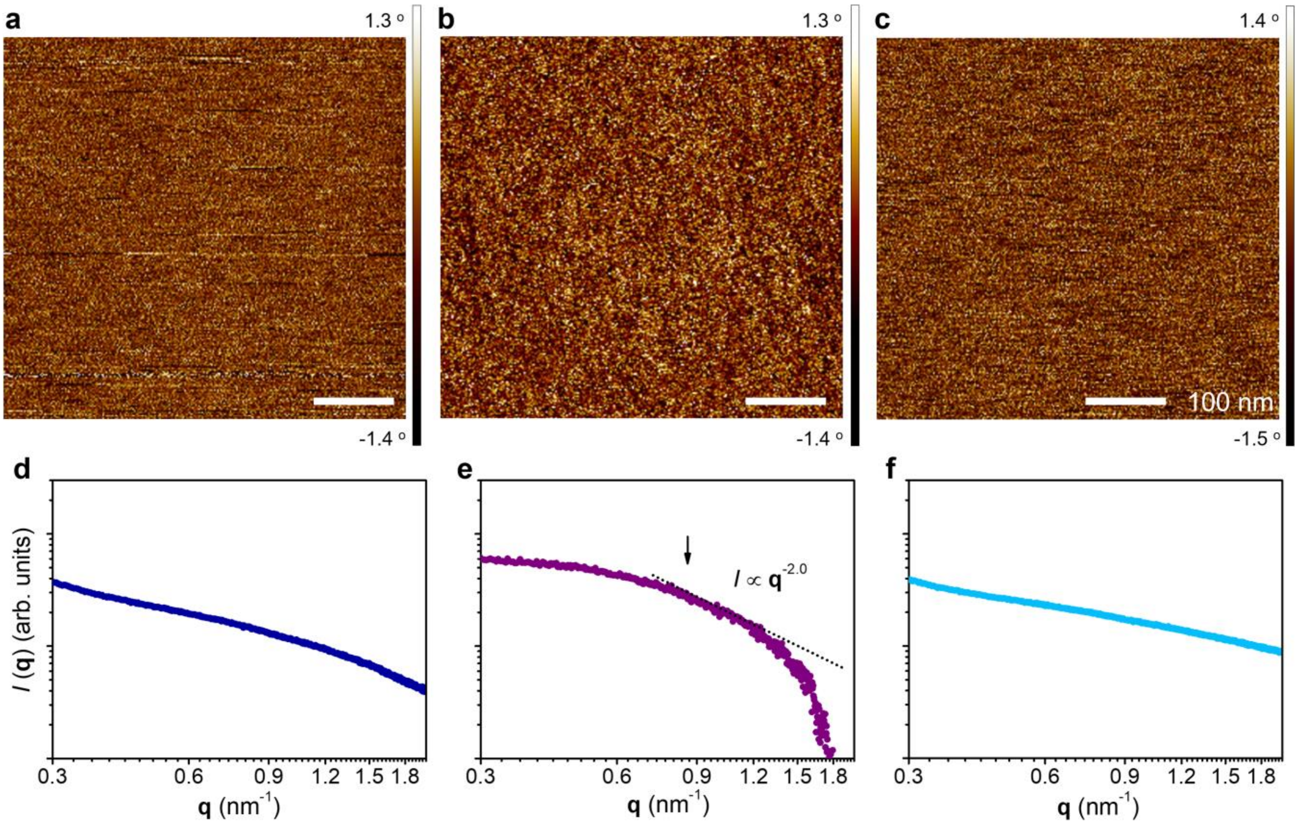

Supplementary Figure 2. Nanostructures of the materials with different molar ratios. AFM phase images of the materials with the molar ratio of (a) 1:1:0, (b) 4:4:1, (c) 5:5:4 (PDMAPS: PAA: IL). Corresponding SAXS plots of the materials with the molar ratio of (d) 1:1:0, (e) 4:4:1, (f) 5:5:4 (PDMAPS: PAA: IL).

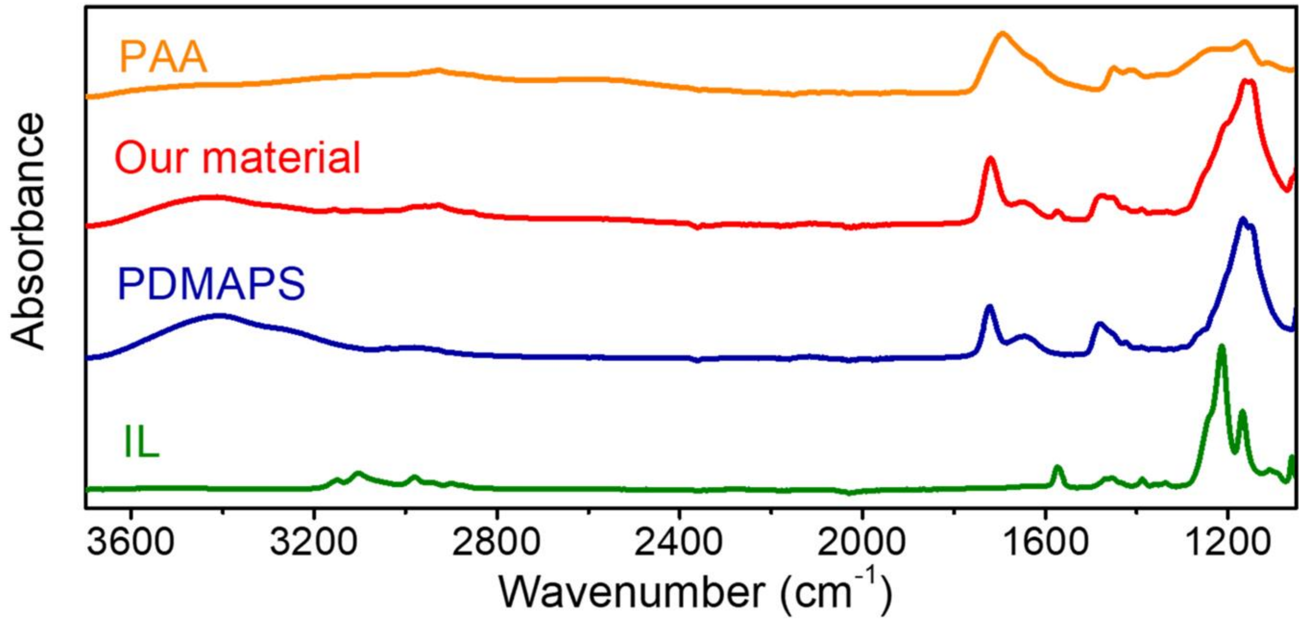

Supplementary Figure 3. IR spectra of the conductor, PAA, PDMAPS, and IL. IR spectra of the intrinsically stretchable conductor, PAA, PDMAPS, and IL in the region of 3700-1050 cm^{-1}.

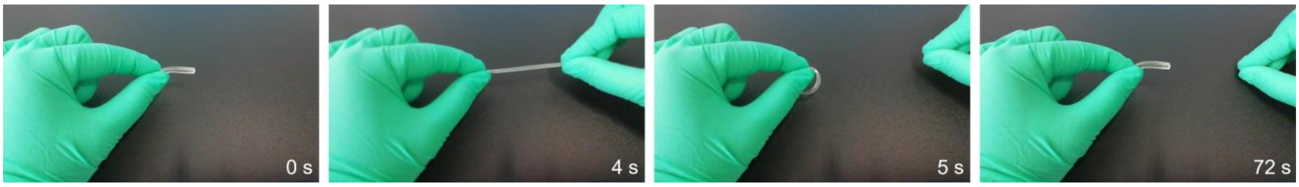

Supplementary Figure 4. The elasticity and recoverability of the stretchable conductor. Photographs of the intrinsically stretchable conductor during a manual stretch-release process.

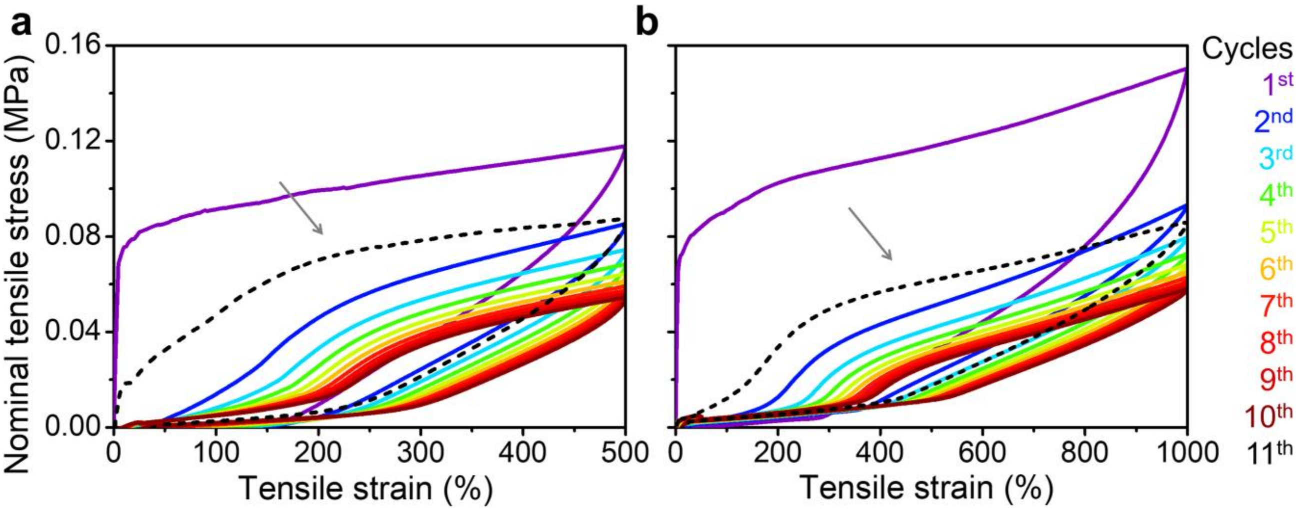

Supplementary Figure 5. Cyclic stress-strain curves of the stretchable conductor. Nominal stress-strain curves of the intrinsically stretchable conductor under cyclic loading in the strain ranges of (a) 500% and (b) 1000%. The black dash lines indicated by the arrows are the stress-strain curves of the stretchable conductor after 10 cycles of the continuous stretching and a resting period of 1 hour. The strain rate is 0.17 s^{-1}.

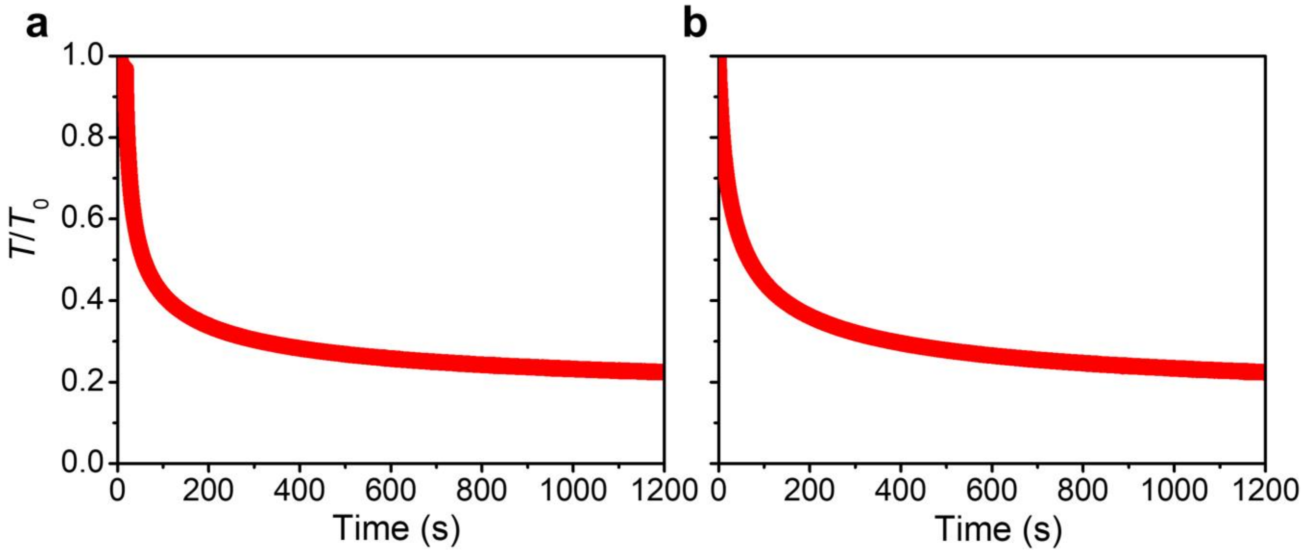

Supplementary Figure 6. Stress-relaxation curves of the stretchable conductor. Normalized stress-relaxation analysis of the intrinsically stretchable conductor after being stretched to (a) 500% and (b) 1000% strain. The stretching rate is 0.17 s^{-1} during the stretching process.

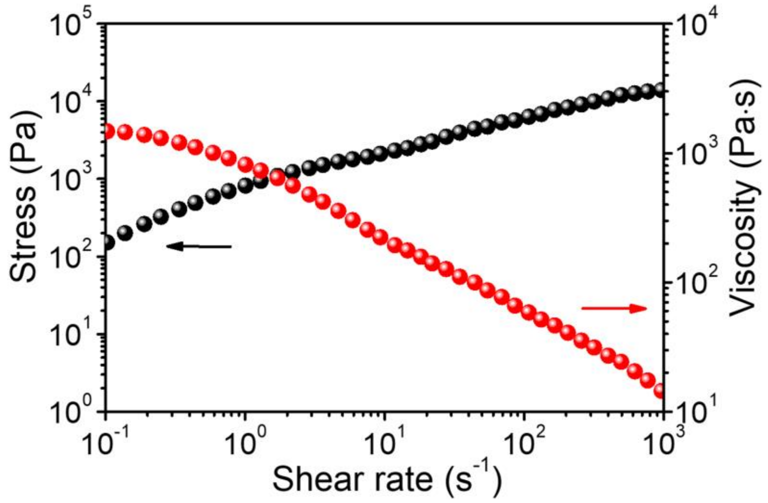

Supplementary Figure 7. Rheology behavior of the precursor ink. Shear-thinning behavior of the precursor of the conductor.

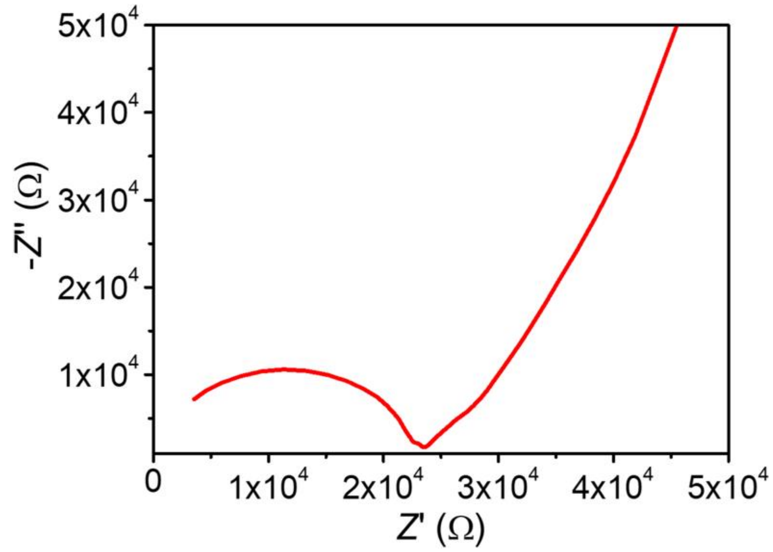

Supplementary Figure 8. The Nyquist plot of impedance spectra of the conductor. The intrinsically stretchable conductor is with the optimized nanostructure (the molar ratio of the PDMAPS, PAA and IL is 2:2:1) in ambient condition.

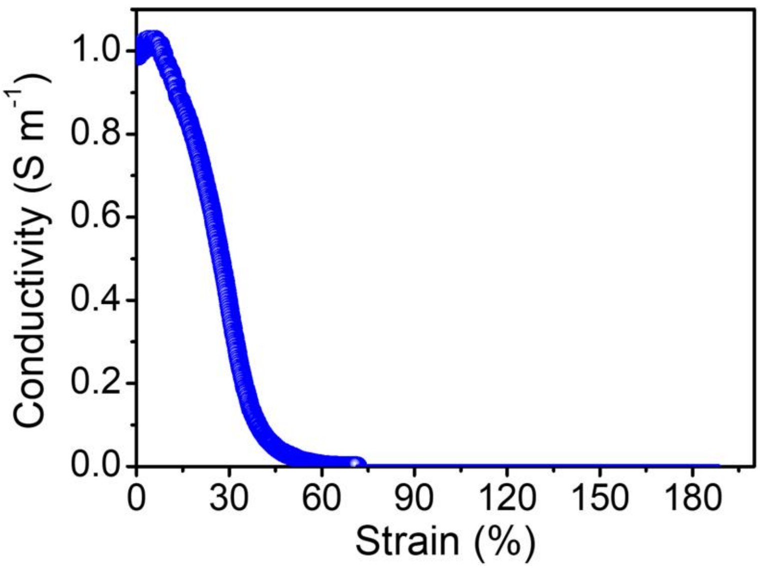

Supplementary Figure 9. Comparison of the stability during stretching. Tensile stress–strain curve of the polydimethylsiloxane/graphene composite at a strain rate of 0.013 s^{-1}. The electronic conductor breaks below 200% strain.

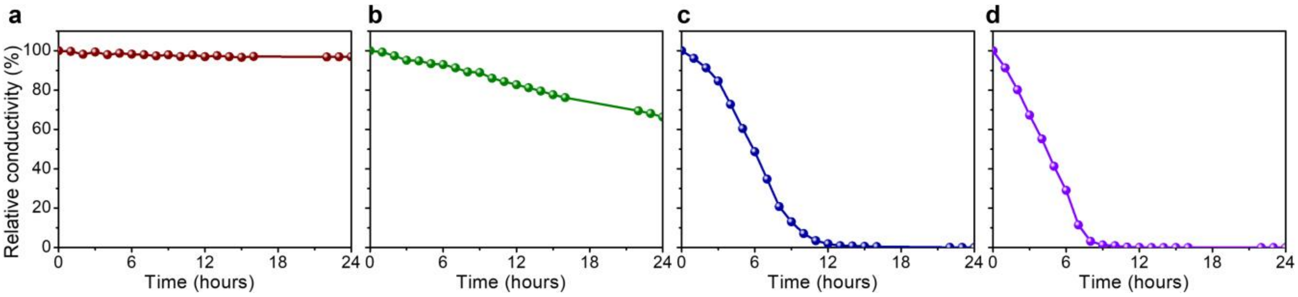

Supplementary Figure 10. Comparisons of the stability at ambient condition. (a) The relative conductivity changes of our material at ambient condition (25 ^{o}C, 60% RH). (b) The relative conductivity changes of a PAA/IL ionogel at the same condition. The decrease of the conductivity may be due to the leakage of IL. (c) The relative conductivity changes of a polyacrylamide/NaCl hydrogel at the same condition. The decrease of the conductivity is ascribed to the loss of free water. (d) The relative conductivity changes of a Ca-alginate hydrogel at the same condition. The decrease of the conductivity is ascribed to the loss of free water.

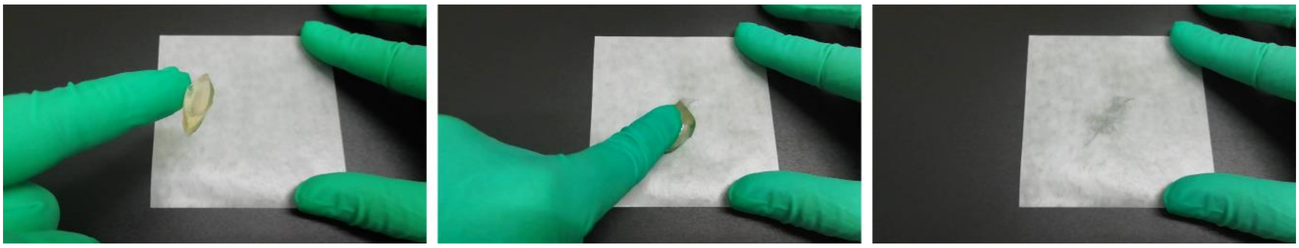

Supplementary Figure 11. Squeezing test of a PAA/IL ionogel. Photographs of a PAA/IL ionogel being squeezed on a plain paper and the leaking IL on the plain paper.

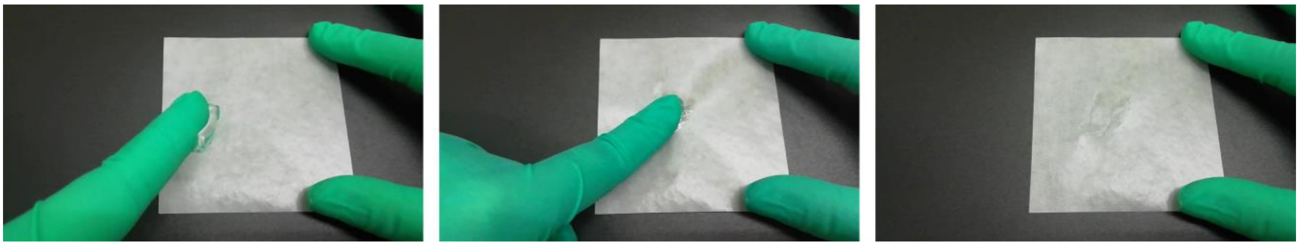

Supplementary Figure 12. Squeezing test of a polyacrylamide/NaCl hydrogel. Photographs of a polyacrylamide/NaCl hydrogel being squeezed on a plain paper and the leaking water on the plain paper.

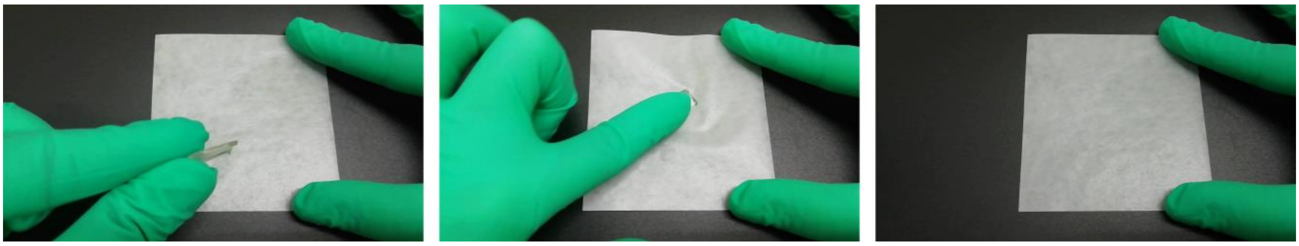

Supplementary Figure 13. **Squeezing test of our material.** Photographs of our material being squeezed on a plain paper and no leaking liquid on the plain paper.

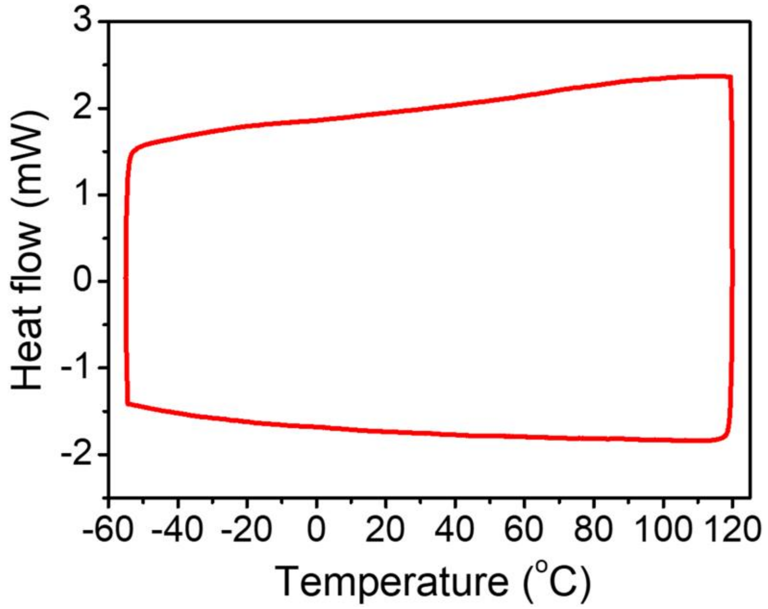

Supplementary Figure 14. **DSC curves of the conductor.** DSC curves in a temperature range of -55 to 120 ^{o}C.

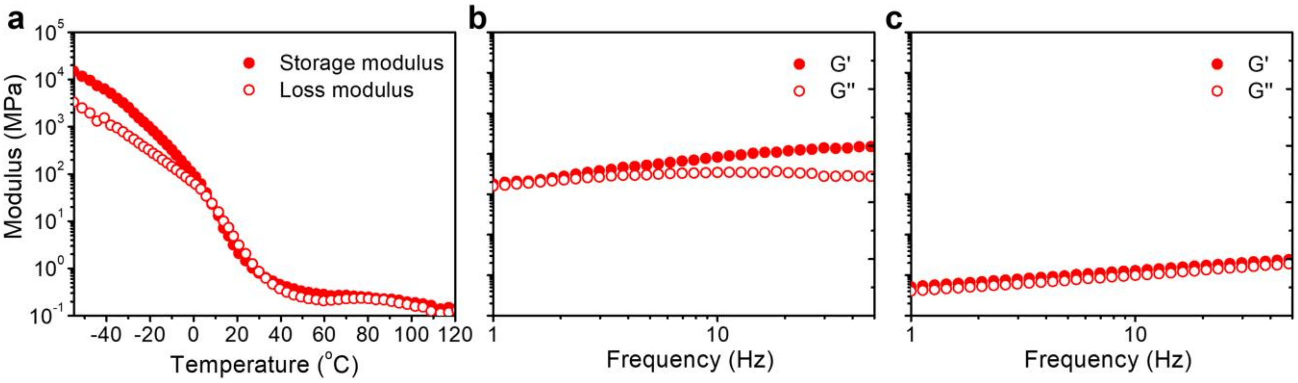

Supplementary Figure 15. **DMA curves and rheological behaviors of the conductor.** (a) DMA curves of the intrinsically stretchable conductor in a temperature range of -55 to 120 ^oC. (b) The rheological behavior of the conductor at the low temperature of -10 ^oC. (c) The rheological behavior of the conductor at the high temperature of 100 ^oC.

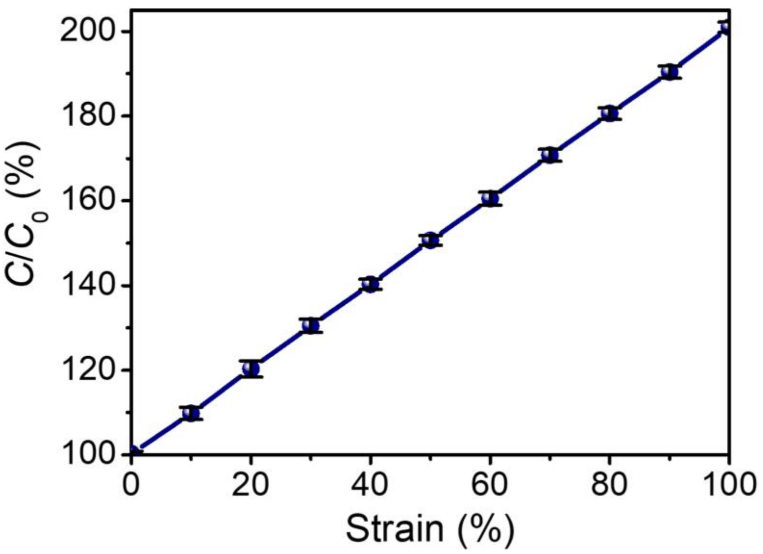

Supplementary Figure 16. The capacitance-strain curve at different temperatures. The capacitance-strain curve of the sensory system in Figure 5. Data are derived from average values of capacitance at different temperatures in Figure 5g, and error bars are very small which confirm the capacitive response is insensitive to the temperature variation (error bars: standard deviations).

Supplementary Table 1. The polarity of different liquids. The polarity of different liquid molecules (at 25 ^{o}C).

	H_2O	DMF	Ethanol	Acetone	n-hexane
Polarity (dielectric constant, $F\ m^{-1}$)	80	38.3	24.3	20.7	Nonpolar

Supplementary Notes

Supplementary Note 1. DFT calculation details

All of the calculations have been carried out at the ωB97X-D level using the 6-31g(d) basis set which includes polarization functions on all the atoms except the hydrogens.[1] Geometries of the PDMAPS, IL, PAA and their complexes have been optimized using Berny's algorithms.[2] A single-point frequency calculation was followed to ensure that the final structure obtained without imaginary frequency. All of the calculations were preformed using the GAUSSIAN 09 package of programs.[3]

Supplementary Note 2. Rheological analysis of the 3D printing process

To analyze the rheological behavior of the precursor during 3D printing, we measured stress and viscosity as a function of shear rates. The viscosity shows shear-thinning behavior with $\eta \approx 1475$ Pa s at low shear rates (0.1 s^{-1}) and $\eta \approx 14$ Pa s at high shear rate (1000 s^{-1}). In this region (0.1-1000 s^{-1}), a power-law fluid model is applied to describe the shear stress as a function of shear rates,

$$\tau = K \cdot \dot{\gamma}^n \text{ ... (1),}$$

in which n is the exponent and K is the consistency index. By performing a power-law fit, the shear-thinning exponent n is calculated to be about 0.46. When the power-law fluid flowing through a cylindrical tube of radius r, the Rabinowitsch–Mooney equation,[4]

$$\dot{\gamma} = (\frac{3n+1}{4n})\frac{4Q}{\pi r^3} \text{ ... (2),}$$

in which Q is the volumetric flow rate, is utilized to predict the shear rate $\dot{\gamma}$, which is about 40 s^{-1}. Therefore, the precursor is printed at the viscosity of about 100 Pa s, and its viscosity increases to > 1475 Pa s immediately after extrusion (assuming a shear rate of far below 0.1 s^{-1}) which allows for shape retention.

Supplementary Note 3. Comparison of the conductivity measured by different methods

We compare the conductivity measured by different methods. When the conductivity is measured by an LCR meter, the applied voltage is 1 V and the measuring frequency is 1 kHz. The conductivity (σ) is calculated by,

$$\sigma = L/RA \text{ ... (3),}$$

in which R is the resistance, L and A are the materials' geometry factors corresponding to the length and cross-sectional area, respectively. At ambient condition, the calculated

conductivity of the conductor with the optimized nanostructure (the molar ratio of the DMAPS, AA and IL is 2:2:1) is 1.2×10^{-2} S m^{-1}. When the conductivity is measured in the same environment by a four electrode method using AC impedance spectroscopy between 0.1 MHz and 1 Hz on a CHI660D electrochemical workstation with potentiostat control, an intersection of the Nyquist plot with the real axis corresponds to resistances of the material. The calculated conductivity is 9.7×10^{-3} S m^{-1}. Therefore, the conductivity of our material measured by different methods is about 10^{-2} S m^{-1} in ambient condition. Unless otherwise stated, we measure the conductivity (resistance) by using the LCR meter for the ease of operation.

Supplementary Note 4. Preparation of electronic and ionic conductors for comparisons

The electronic conductor for the comparison has been reported in our previous work.[5] In brief, the polydimethylsiloxane/graphene composite was prepared by mixing 50 mL dimethylbenzene, 2 g polydimethylsiloxane with curing agent (Sylgard184), and 14 wt% graphene (The Sixth Element (Changzhou, about 1 μm diameter) Materials Technology Co., Ltd.). After 1 hour of ultrasonic treatment, the mixture was subjected to solvent evaporation for 24 hours and further curing at 65 ^{o}C for 2 hours. The stretchable electronic conductor was finally obtained.

The polyacrylamide/NaCl hydrogel (a chemically crosslinke hydrogel) was prepared by radical polymerization of 2.2 M acrylamide monomer in the mixed aqueous solution of 1.32 mM N,N'-methylenebis(2-propenamide) and 4 M NaCl, which was initiated by 1 mM APS and carried out at 70 ^{o}C for 6 h.[5]

The PAA/IL ionogel (with the approximate conductivity and the same IL, 1-ethyl-3-methylimidazolium ethyl sulfate) was prepared by radical polymerization of 2.5 mL AA monomer and 10 μL PEGDA in the IL (7.5 mL). The polymerization was initiated by 0.0122 g APS and carried out at 70 ^{o}C for 6 h.

The Ca-alginate hydrogel (a physically crosslinked hydrogel) was prepared by the gelation of 10 wt% alginate solution (15 mL) with the addition of 5 mL of 0.1 M $CaCl_2$ solution at room temperature for 2 days.

Supplementary Note 5. DSC, DMA results and rheological behaviors of the ionic conductor

There is no melting point observed in DSC curves of this ionic conductor, which indicates there is no free water and the IL in our material does not crystallize even at the temperature

as low as -55 ^{o}C. Thus the IL is effectively bonded by polymer networks with good environmental stability. Interestingly, there is no glass transition observed in the DSC curves, while a very broad glass transition region is shown in DMA curves, similar to that of a commercial perfluorosulphonic acid ionomer (Nafion), which also shows no glass transition in DSC curves but a quite broad glass transition range in DMA curves.[6] It is worthwhile to note that, the glass transition temperature in DMA curves is determined by the shapes of the curves of storage modulus and the loss modulus.[6,7] There is no plateau region of the storage modulus curve and no peak of the loss modulus curve in our material. They suggest that the polymers' local segmental motion is not completely frozen even at the temperature as low as -55 ^{o}C. In another word, there are always different modes of molecular motion that are active in such a broad region. Furthermore, as shown in the rheological results, the polymer networks maintain the solid-like elasticity at -10 and 100 ^{o}C, since the storage modulus (G') is always higher than the loss modulus (G''). Therefore, this material is anti-freezing and also stable at high temperature, confirmed by the DSC, DMA and rheology measurements.

Supplementary Note 6. Strain sensing analysis

The capacitive strain sensing is based on the mechanism of parallel-plate capacitance,

$$C = \varepsilon S/4\pi k d \text{ ... (4),}$$

in which C, ε, k, S and d are the capacitance, dielectric constant of the dielectric layer, electrostatic constant, effective area of the conductive layer, and the thickness of the dielectric layer. Assuming that the volume and permittivity of the sensory system remain constant, when it is stretched by a factor, $\lambda = L/L_0$, the area ($S = W\ L$) of the conductive layer is scaled by a factor of $\sqrt{\lambda}$, and the thickness of the dielectric layer (d) is scaled by a factor of $\frac{1}{\sqrt{\lambda}}$. Consequently, the capacitance C scales as $C = C_0\,\lambda$ (C_0 is the initial capacitance).

Supplementary Note 7. Pressure sensing analysis

The capacitive pressure sensing also depends on the dimension change of parallel-plate capacitance. Since the intrinsically stretchable conductor has a much higher modulus than the dielectric layer (the compression modulus of the intrinsically stretchable conductor and VHB layer is about 1000 and 60 kPa, respectively), the capacitance change of the sensory system is mainly determined by the dimension change of the dielectric layer. When the sensory system is compressed by a factor of k, the thickness of the dielectric layer is scaled

by a factor of $1-k$. As a result, the capacitance C scales as $C = C_0\frac{1}{1-k}$, and the pressure sensitivity is related with the modulus (*E*) of the VHB layer.

Supplementary Note 8. Thermodynamic analysis of the self-powered humidity sensing

Moisture promotes the ionization of one side of the intrinsically stretchable conductor, in which more free ions are released, and the gradient distribution of ions enables a diffusion process which can be described as:

$$J_{dif} = -qD\frac{dc}{dx} \text{ ... (5),}$$

in which J_{dif}, q, D, c and x are diffusion current density, electric quantity of elementary charge, diffusion coefficient, concentration of ions and distance to the surface exposed to moisture, respectively. When the ions migrate and reach the other side, the drift induces electric field between two sides of the conductor which can be defined by:

$$J_{dri} = \sigma E_i \text{ ... (6),}$$

where J_{dri}, σ and E_i are drift current density, ionic conductivity and induced electric field intensity, respectively. When the thermodynamic equilibrium is established, *i.e.*,

$$J_{dif} + J_{dri} = 0 \text{ ... (7),}$$

the induced electric field intensity can be described as:

$$E_i = \frac{qD}{\sigma}\cdot\frac{dc}{dx} \text{ ... (8).}$$

Therefore, the induced voltage V_i perpendicular to the surface of the conductor, can be described as:

$$V_i = \int\frac{qD}{\sigma}\cdot\frac{dc}{dx} \text{ ... (9).}$$

Ideally, each part of the conductor is supposed to have the same diffusion coefficient and ionic conductivity which are irrelevant with the distance x, and hence the induced voltage V_i can be expressed as:

$$V_i = \frac{qD}{\sigma}\int\frac{dc}{dx} \text{ ... (10),}$$

suggesting that the intensity of induced voltage directly corresponds to the gradient of ions.

Supplementary References

1 Cao, Y. *et al.* A transparent, self-healing, highly stretchable ionic conductor. *Adv. Mater.* **29**, 1605099 (2017).

2 Schlegel, H. B. Optimization of equilibrium geometries and transition structures. *J. Comput. Chem.* **3**, 214-218 (1982).

3 Frisch, M. J. T., G. W.; Schlegel, H. B.; Scuseria, G. E.; Robb, M. A.; Cheeseman, J. R.; Scalmani, G.; Barone, V.; Mennucci, B.; Petersson, G. A. Gaussian 09 Revision A.1. Gaussian Inc. *Gaussian 09 Revision A.1. Gaussian Inc* (2009).

4 Tian, K. *et al.* 3D printing of transparent and conductive heterogeneous hydrogel–elastomer systems. *Adv. Mater.* **29**, 1604827 (2017).

5 Lei, Z. & Wu, P. Adaptable polyionic elastomers with multiple sensations and entropy-driven actuations for prosthetic skins and neuromuscular systems. *Mater. Horiz.* **6**, 538-545 (2019).

6 Xie, T. Tunable polymer multi-shape memory effect. *Nature* **464**, 267 (2010).

7 Lei, Z. *et al.* The proper glass transition temperature of amorphous polymers on dynamic mechanical spectra. *J. Therm. Anal. Calorim.* **116**, 447-453 (2014).
