# Supplementary material for: A highly transparent and ultra-stretchable conductor with stable conductivity during large deformation
Source: Nat Commun. 2019 Jul 31;10:3429. doi: 10.1038/s41467-019-11364-w (PMC6668389; doi:10.1038/s41467-019-11364-w)
Supplement: Supplementary file 2 — Description of Additional Supplementary Files [file 41467_2019_11364_MOESM2_ESM.pdf]

Description of Additional Supplementary Files

File Name: Supplementary Movie 1
Description: A movie showing the elasticity and recoverability of this conductor.

File Name: Supplementary Movie 2
Description: A movie showing the squeezing test of a PAA/IL ionogel.

File Name: Supplementary Movie 3
Description: A movie showing the squeezing test of a polyacrylamide/NaCl hydrogel.

File Name: Supplementary Movie 4
Description: A movie showing the squeezing test of this conductor.

File Name: Supplementary Movie 5
Description: A movie showing the transparent integrated sensory system can track the movements of a robotic finger with real-time capacitance signals.

File Name: Supplementary Movie 6
Description: A movie showing the transparent integrated sensory system can sense temperature stimulus with real-time resistive response.

File Name: Supplementary Movie 7
Description: A movie showing the transparent sensory system can recognize H2O.

File Name: Supplementary Movie 8
Description: A movie showing the transparent sensory system can recognize ethanol.
